# A Comparative Study on Efficacy of Negative Pressure Wound Therapy Versus Standard Wound Therapy for Patients With Compound Fractures in a Tertiary Care Hospital

**DOI:** 10.7759/cureus.23727

**Published:** 2022-04-01

**Authors:** Arun Kumaar, Arun H Shanthappa, Prabhu Ethiraj

**Affiliations:** 1 Department of Orthopedics, Sri Devaraj Urs Medical College, Sri Devaraj Urs Academy of Higher Education and Research, Kolar, IND

**Keywords:** standard wound therapy, trauma, angiogenesis, compound fracture, vacuum assisted closure, negative pressure wound therapy

## Abstract

Background: Orthopedic surgeons face a difficult task in treating serious open fractures, which usually result in complications, morbidity, and even amputation. Compound fracture wounds were traditionally treated with a standard saline dressing. To avoid infection and problems during therapy, several studies found that open fractures require early skeletal stability as well as soft tissue repair. In favoring the above fact vacuum-assisted closure (VAC) is now undergoing a paradigm shift. With this background, this study aimed to compare the effects of VAC dressing versus standard wound dressing on compound fracture wounds.

Methodology: This study has been conducted as a single-blind randomized control trial among 128 patients who got admitted to the Department of Orthopedics, R L Jalappa Hospital attached to Sri Devaraj Urs Medical College, Karnataka, India from August 2019 to November 2021. The study participants were randomly allotted into two groups negative pressure wound therapy (NPWT) and standard wound therapy (SWT) consisting of 64 participants in each group. VAC dressing was used on group NPWT, while normal saline wound dressing was used on group SWT. Both groups were followed up for a month after their discharge from the hospital. Frequency in dressing changes wound healing time, infection presence, and hospitalization days were all recorded and compared over one month. The data collection was done using questionnaires and the statistical analysis was done with SPSS version 21 (Chicago, IL: IBM Corp.).

Results: There was a statically significant difference favoring group NPWT compared to group SWT with a hospital stay, number of dressings required, wound size reduction, wound healing time, and deep infection rate (p<0.05).

Conclusions: The use of NPWT speeds up the healing of compound fracture wounds significantly. It is affordable and can be used as a substitute in resource-constrained areas to reduce infection and manage open fracture wounds quickly.

## Introduction

Orthopedic surgeons face a difficult task in treating serious open fractures, which usually result in complications, morbidity, and even amputation. Open fractures are associated with high-impact trauma. In the management of compound fractures, wound therapy and infection control are essential parts. These fractures are coupled with a higher risk of infection and complications during therapy, so it's critical to get them managed without more ado. It is a fact that severe open fractures were presented with a range of 25-66% infection rate [1-3]. Several determinants are always to be well-thought-out when assessing and treating compound fractures in the extremities, including the patient's condition, fracture type, antimicrobial therapy, wound debridement, site and size of the wound, neurovascular status, and the degree of muscle tear [4,5]. Usually, the Gustilo-Anderson classification model is used to classify the compound fractures [6].

The infection rate in complex fractures was shown to be 0-2% in type I fractures, about 2-10% in type II fractures, and it rises to 10-50% in type III fractures. It is found that this infection is not caused by the duration of antimicrobial therapy or the time it takes for the wound to heal [1,7]. Negative pressure wound therapy (NPWT) is a type of management of wounds after surgery, which is done using foam or gauze under negative pressure to assist the wound healing. Vacuum-assisted closure (VAC) is the most often used NPWT [8-10]. It was first introduced by Morykwas et al. about two decades ago [11]. In the above technique, negative pressure is applied to the wound using a particular closed wound dressing that is applied continuously or intermittently. And this results in a biological impact that improves wound healing [8]. NPWT was first recognized as an adjuvant therapy that aids in the healing of difficult-to-heal complex wounds [12]. After that many studies and clinical trials gave supporting evidence about this method for wound healing in treating compound fractures [12-16]. Moreover, it is not only an effective method that improves wound healing, it is also a cost-effective method that most patients can afford. And It becomes a treatment guideline in many institutions nowadays [1,2]. The mechanism of action of the VAC device is as follows - the first step includes drawing the wound edges together by wound debridement [7,17]. Then applying negative pressure wound therapy stabilizes the wound from any added infection. Once the granulation tissue has appeared, the patients proceeded with secondary procedures like skin grafting for closure of the wound [9,10,17]. Thus, NPWT procedure facilitates the skin grafting process [18]. Despite the introduction of new therapeutic strategies for improved wound care, such as dressings, local growth factors, hyperbaric oxygen, and local and systemic antiseptic agents, continuous wound management remains a clinical problem [1]. Data from various research show that negative pressure dressings provide a sufficient environment for open fracture to heal because it prevents the site from infection which hinders the healing process and decreases wound complications [7,18]. This study aimed to compare the effects of VAC versus standard wound dressing on open-compound fracture wounds.

## Materials and methods

This study was a single-blinded randomized control study. All the study participants (taken by universal sampling) who were admitted to the Department of Orthopedics, R L Jalappa Hospital attached to Sri Devaraj Urs Medical College, Karnataka, India, for a period of 15 months (August 2019 to November 2021) were included in this study (n=128). The ethical clearance was obtained from the institutional ethical committee with approval number DMC/KLR/IEC/445/2021-22.

Inclusion and exclusion criteria

About 128 individuals participated in the study. Patients of age 30-70 years, who had a compound fracture and underwent meticulous wound debridement were included. The patients with a history of orthopedic surgery, fractures associated with other fractures, pregnancy, the need for vascular surgery, malnutrition, any dermatological conditions, those already on immunosuppressive therapy, and previous osteomyelitis were excluded from the study.

Data collection

After getting the informed consent, patients were randomized into group NPWT and group SWT by using the envelope method. The treatment allocations were generated randomly and sealed in consecutively numbered indistinguishable envelopes. The random sequence for these blocks was generated using a generator that gives random numbers using the SPSS software version 21 (Chicago, IL: IBM Corp.). All participant's fracture types were classified using the Gustilo-Anderson classification system and type II, type III A, type III B, and type III C wound types were included in the study. The vacuum-assisted closure technique was used to administer negative pressure wound therapy to patients in group NPWT (VAC). We got a clean wound after meticulous debridement of the open fractures and sponge foam was applied to the wound. Then, the wound was enclosed with an adhesive blind. Finally, the device's outer end was attached to the suction tube's inner end, which was introduced into the dead wound space. Wound dressings were changed every 72 hours, and negative pressure was maintained for two weeks. For five minutes on, two minutes off, the pressure was maintained at -125 mm Hg intermittently. Patients in Group SWT had saline-soaked wound dressing with gauze twice a day. Patients were tracked for a month after they were discharged. Patients were encouraged to return to the hospital for routine check-ups after release, and all participants were tracked throughout the trial. the patients were followed up for every week until complete healing is achieved by VAC method or secondary method like skin grafting. If there was any granulation tissue, if the wound bed had turned redder, if the wound drainage had shrunk, and if the wound's dimensions had shrunk then this indicates wound healing. When a sufficient granulation base was developed, intervention therapy was stopped. To determine the existence of infection, a culture and sensitivity of the wound were performed before and after treatments. Based on the study by Gupta et al., a minimum sample size of 64 per group was calculated by replacing in the below formula, the meantime of wound healing as a key variable, assuming a 95% confidence interval and an 80% power level, as well as the possibility of dropouts [19].

$n=\frac{2S_{p}^{2}[Z_{1-\alpha/2 }+Z_{1-\beta }]^{2}}{\mu _{2}^{d}}$

$S_{p}^{2}=\frac{S_{1}^{2}+S_{2}^{2}}{2}$

For quantitative variables, the data were given as mean standard deviation, while for qualitative factors, it was presented as percentages. For categorical variables, Fisher's exact test and chi-square test were employed, while for continuous variables, paired/unpaired t-test was utilized. Statistical significance in the results was defined as a p-value of less than 0.05 and SPSS software version 21 was used for data analysis. From an ethical standpoint, the participants' dignity and well-being were always preserved. The researcher acquired the participants' permission to use their true identities in the research report, and the research data remained confidential throughout the study. The study is self-funded with the help of the institution.

## Results

In this study, 128 patients were enrolled with compound fractures for management. This included 33 (25.78%) females and 95 (74.22%) males. The study participant’s average age was 43.32±8.56 years, ranging from 30 to 70 years. There was no significant difference between the two intervention groups in terms of demographic details collected such as age, sex, weight, and height. The mean number of stays in hospital was statistically significant between NPWT and SWT groups (9.55±2, 22 vs 11.67±2.98 days) (p<0.001) (Table 1).

**Table 1:** Basic demographics of patients. *Statistically significant. NPWT: negative pressure wound therapy; SWT: standard wound therapy; M/F: male/female

Variable	Group NPWT	Group SWT	p-Value
Age (years)	44.74±13.70	41.52±10.89	0.144
Gender (M/F)	44/20	45/19	0.793
Height (cm)	153.52±27.06	152.27±37.39	0.829
Weight (kg)	59.58±13.44	61.19±13.94	0.507
Mean duration of hospital stay	13.55±2,22	20.67±2.98	<0.001*

The mean number of dressings per patient was noted as significantly lower in the NPWT group (4.32) than in the control group (15.77) (p<0.001). Wound healing time was measured to be significantly lower, 17.45±3.33 days, in the NPWT group and 32.76±3.88 days in the SWT group (p=0.001). The prevalence of infection was observed at the end of the study in both the comparison groups. It was observed that in the VAC dressing group, there were four cases of deep infection and in the standard dressing group, there were 13 cases with deep infection. Eight patients in group NPWT and nine patients in group SWT needed skin grafts. One patient in group NPWT and two patients in group SWT needed flap procedures (Table 2). The patterns of fractures type are shown in Table 3.

**Table 2:** Wound characteristics of patients. *Statistically significant. NPWT: negative pressure wound therapy; SWT: standard wound therapy

Variable	Group NPWT	Group SWT	p-Value
Mean number of dressings	4.32±0.27	15.77±0.44	<0.001*
Mean wound healing time (days)	17.45±3.33	32.76±3.88	<0.001*
Acute wound infection	0(0.00)	2(3.13)	0.154
Deep infection (n %)	4 (6.25)	13 (20.31)	0.019*
Delayed closure	55 (85.94)	53 (82.81)	0.626
Skin graft	8 (12.50)	9 (14.06)	0.795
Flap	1 (1.56)	2 (1.56)	0.559

**Table 3:** Distribution of fractures by grading. NPWT: negative pressure wound therapy; SWT: standard wound therapy

Variable	Group NPWT	Group SWT	p-Value
Grade I	0 (0.00)	0 (0.00)	0.601
Grade II	6 (9.38)	11 (17.19)	
Grade III A	23 (35.94)	19 (29.69)	
Grade III B	34 (53.13)	33 (51.56)	
Grade III C	1 (1.56)	1 (1.56)	

The size of wounds in the two groups was similar. Mean area of the wound was 211 cm sq in NPWT group compared to 212 sq cm in SWT group. After the intervention period, the average area of the wound was observed as 122 cm sq in NPWT group compared to 145 sq cm in SWT group. Wound surface reduction difference was found to be statistically significant before and after treatment group NPWT compared to group SWT (p=0.001) (Table 4).

**Table 4:** Distribution of wound area before and after intervention. *Statistically significant. NPWT: negative pressure wound therapy; SWT: standard wound therapy

Mean area of the wound (sq cm)	Group NPWT	Group SWT	p-Value
Baseline	211.33±24.67	212.76±22.56	0.733
End line	122.15±13.49	145.88±15.78	<0.001*

Describing two individual cases

**Figure 1:**
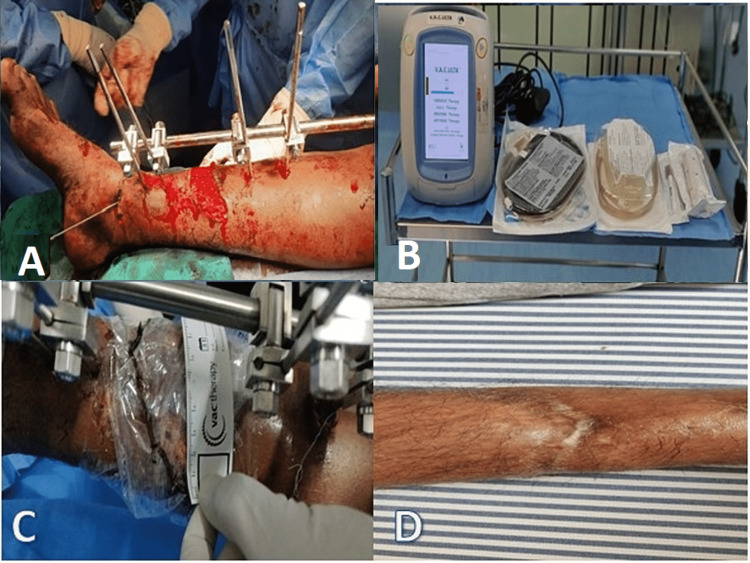
Negative pressure wound therapy of the patient and the application of vacuum-assisted closure device. The images show (A) external fixator application, (B) vacuum-assisted closure device, (C) the application of negative pressure wound therapy by vacuum-assisted closure device, and (D) healed wound post autologous skin grafting.

A 52-year-old man was brought to the emergency room following allegedly being injured in a motorcycle collision. His vitals were stabilized after the initial triage. Blood tests and x-rays were done and diagnosed with compound fracture distal third right tibia (Gustilo-Anderson type III B). The patient was taken to the operating room and wound debridement, fracture reduction, and fixation were done using an external fixator (Figure 1A). VAC dressing was applied to the open wound in the same setting and negative pressure was administered at 125 mmHg intermittently (Figures 1B, 1C). The dressing was changed every three days for a period of two weeks. On day 15, an autologous skin graft was performed, and the patient was discharged after a week and followed up on an outpatient basis. On follow-up day 30, wound had healed satisfactorily (Figure 1D).

**Figure 2:**
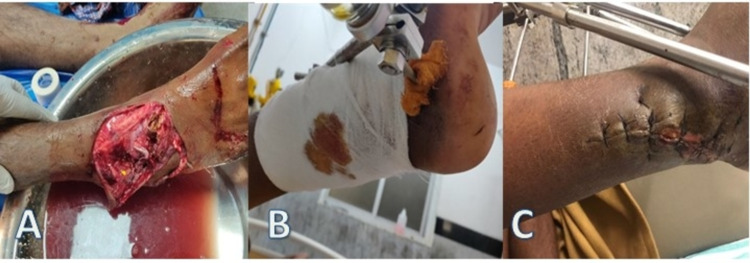
Standard wound therapy of the patient with saline dressing. The images show (A) compound fracture-dislocation of right ankle joint, (B) saline-soaked dressing with external fixator present in situ, and (C) approximation of wound following secondary suturing of the wound.

A 34-year-old man was brought to the emergency room after allegedly being involved in a traffic collision. His vitals were stabilized after the initial triage. Following standard blood tests and x-rays, diagnosed as compound fracture-dislocation of right ankle joint (Figure 2A). The patient was taken to the operating room. Wound debridement, open reduction of ankle dislocation, and fracture fixation were done using an external fixator. For a period of four weeks, saline-soaked wound dressing was done twice a day and wound was approximated by secondary suturing (Figures 2B, 2C). The wound had healed satisfactorily and the patient was followed up on an outpatient basis.

## Discussion

In the present study, the management of open fractures was done by two methods namely VAC dressing or standard dressing thus, dividing study participants into two groups. There was no statistically significant difference observed between the two groups regarding basic demographic variables like age, gender, height, and weight. Fewer studies have stated similar results when basic demographic variables were matched between intervention groups [20,21].

The mean number of stays in the hospital was shorter in NPWT group patients compared to the SWT group patients by seven days. The results obtained in our study were similar to the results of Kaushik et al. who found a 27% reduction in mean hospital stay compared to 34% in our study [16]. It can be assumed that the use of NPWT encourages faster soft tissue coverage resulting in a lesser number of inpatient days.

The mean number of dressings in NPWT group patients was lower compared to the SWT group patients by 11 dressing episodes. The results obtained in the present study were comparable with the results of Kaushik et al. who found an 84% reduction in the mean number of dressings compared to 73% in our study [16]. This is because NPWT creates a positive wound environment by reducing edema, increasing blood flow, reducing healing inhibitors, and promoting granulation causing lowering wound exudate volumes.

The average time required for wound healing was quicker in NPWT group patients compared to the SWT group patients by 15 days. The finding obtained in the present study were comparable with the results of some studies which found a 35-54% reduction in mean wound healing time compared to 47% in our study [10,15,22]. Maintaining hemostasis, reducing inflammation, dominant fibroblast activity, renewal of collagen fibers, and contraction of the wound through the activity of myofibroblasts are the basic principles of NPWT, thus facilitating quicker wound healing time.

The incidence of deep infection was lower in NPWT group patients compared to the SWT group patients by 14 percentage points. The finding obtained in the present study were comparable with the results of Costa et al. who found a 69% reduction in deep infection incidence compared to 56% in our study [20]. Proper application of NPWT results in near-complete debridement and adequate irrigation before wound closure, thus preventing bacterial access to the wound bed. This can remarkably reduce the risk of deep infection.

The mean area of the wound reduced rapidly in NPWT group patients after the treatment period compared to the SWT group patients by 32 sq cm. The results obtained in our study were relevant to the results of Quatman et al. who found a 28% reduction in the mean area of wound compared to 36% in our study [23]. Efficient debridement due to NPWT leads to the removal of devitalized and necrotic tissues. This is critically important to the initiation of healing and faster wound area reduction. 

Limitations

However, there are some limitations of this study, as this trial only addresses the early course of wound healing and limited sample size due to paucity of time and funds. The severity of the injury, smoking, and other comorbid conditions like diabetes which may hinder wound healing are the confounding factors that are not included in this study.

## Conclusions

This study concluded that VAC dressing helps in expediting the rate of wound healing and control of infection in open fractures. NPWT reduced hospitalization days, dressing counts, wound area, healing times, and incidence of deep infection. The ease of application and affordability makes NPWT a viable treatment support option. This study recommends trials on VAC dressing to be conducted on a higher sample of patients with compound fractures preferably in a multi-center approach. Till then clinicians should consider using NPWT over standard treatment for wound care if the healthcare settings permit.
